# Indoleamine 2,3‐dioxygenase gene expression and kynurenine to tryptophan ratio correlation with nasopharyngeal carcinoma progression and survival

**DOI:** 10.1002/iid3.690

**Published:** 2022-08-29

**Authors:** Sameh Souissi, Randa Ghedira, Yosra Macherki, Ahlem Ben‐Haj‐Ayed, Sallouha Gabbouj, Yasmine Remadi, Imen Sfar, Zohra Chadli, Karim Aouam, Mohsen Hassine, Noureddine Bouaouina, Abdelfattah Zakhama, Elham Hassen

**Affiliations:** ^1^ Laboratory of Molecular Immuno‐Oncology, Faculty of Medicine of Monastir University of Monastir Monastir Tunisia; ^2^ Higher Institute of Biotechnology of Monastir University of Monastir Monastir Tunisia; ^3^ Research Laboratory in Immunology of Renal Transplantation and Immunopathology Tunis El Manar University Tunis Tunisia; ^4^ Department of Pharmacology University of Monastir Monastir Tunisia; ^5^ Department of Hematology Fattouma Bourguiba University Hospital Monastir Tunisia; ^6^ Department of Cancerology and Radiotherapy Farhat Hached University Hospital Sousse Tunisia

**Keywords:** indoleamine 2,3‐dioxygenase, nasopharyngeal carcinoma, peripheral blood, progression, survival

## Abstract

**Introduction:**

Indoleamine 2,3‐dioxygenase (IDO) is an immunosuppressive tryptophan‐depleting enzyme expressed in nasopharyngeal carcinoma (NPC) tissue. However, IDO has not been reported in the peripheral blood of NPC patients. The aim of this study was to analyze, IDO1 and IDO2 messenger RNA (mRNA) expression, the kynurenine (Kyn) and tryptophan (Trp) plasma levels, their clinical values and their relationship with cytokine levels in NPC.

**Methods:**

We evaluated IDO1 and IDO2 mRNA expression in peripheral blood mononuclear cells (PBMC) by quantitative real‐time PCR, plasma Trp and Kyn levels by HPLC, and cytokine levels by ELISA in 75 NPC patients and 51 healthy controls.

**Results:**

Compared to controls, IDO1 mRNA expression was significantly upregulated and IDO2 mRNA expression was significantly downregulated in PBMC of patients. Also compared to controls, plasma Kyn levels and Kyn/Trp ratio were significantly higher in patients. At the time of diagnosis, the plasma Kyn/Trp ratio was associated with advanced cancer status and was an independent prognostic factor for worse disease‐specific survival. According to cancer stages, IDO1 mRNA expression was positively correlated with plasma Kyn/Trp ratio in patients with earlier stages (I–II–III) but negatively correlated in patients with the late‐stage cancer (IV). Tumor necrosis factor‐α, interleukin (IL)‐6 and IL‐10 levels were significantly higher in patients compared to controls. Moreover, and despite treatment, patients simultaneously carrying high plasma Kyn/Trp ratio and high plasma IL‐6 and IL‐10 levels at diagnosis died approximately 1 year after first diagnosis.

**Conclusion:**

Measuring blood IDO mRNA expression and Kyn/Trp ratio at diagnosis could be a potential marker to evaluate NPC progression and predict survival outcome.

## Graphical abstract

This study provides evidence for the benefit of measuring blood indoleamine 2,3‐dioxygenase (IDO) mRNA expression and Kyn/Trp ratio, at diagnosis as a potential marker to evaluate nasopharyngeal carcinoma (NPC) progression and predict survival outcome

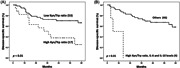

## 1 INTRODUCTION

Nasopharyngeal carcinoma (NPC) is a squamous‐cell carcinoma of the nasopharyngeal epithelium. Globally, NPC is a relatively uncommon malignant tumor, accounting for 0.7% of cancers worldwide and for 0.8% of all cancer deaths.^1^ Its incidence rates are low in most parts of the world (below 1/100,000 persons), and have declined gradually worldwide over the past decades. Nevertheless, NPC is still prevalent mostly in Southeast Asia, Southern China and, North and East Africa.^2^ Tunisia, a Northern African country, is at intermediate risk with a bimodal age distribution and incidence rates of 3.6 and 1.6/100,000 respectively in men and women. Gender‐specific distribution also shows a male predominance in NPC incidence rates with a sex ratio of 2.1. Undifferentiated carcinoma of nasopharynx type (UCNT) is the most common histological type accounting for 93.4%.^3^

The development of NPC is strongly influenced by the host immune system interacting with genetic, environmental and Epstein‐Barr virus‐associated factors.^4^ Despite substantial T cell infiltration, NPC is still potentially invasive with a less favorable prognosis at an advanced stage. In fact, malignancy establishes a number of mechanisms shaping an immunosuppressive microenvironment to disable immune response and support tumor growth.^5^ Indoleamine 2,3‐dioxygenase (IDO) is a major immunosuppressive effector through tryptophan (Trp) metabolism disruption. It is the first and the rate‐limiting enzyme of the kynurenine (Kyn) pathway, which catalyzes the oxidative breakdown of l‐Trp to *N*‐formyl‐kynurenine, which is then rapidly converted into l‐Kyn. IDO exerts important immunosuppressive effects basically by the dysregulation of balance between effector T cells and regulatory T cells (Tregs). Trp depletion suppresses T cell immune response through GCN2 kinase activation and, on the other hand, the production of bioactive Kyn pathway compounds promotes Treg differentiation through the aryl hydrocarbon receptor (AhR) activation.^6^, ^7^ The first studies established a relationship between IDO and immunosuppression by preventing T cell‐mediated rejection of allogeneic conception.^8^ So far, IDO has been proved to be an important mechanism of tumor immune tolerance as well as an attractive target for cancer immunotherapy.^9^ IDO was named IDO1 since an isozyme named IDO2 has been more recently identified.^10^ Both enzymes catalyze the same enzymatic step, but they show different expression and regulation patterns in steady and pathological settings. IDO2 seems to be expressed at basal levels compared to IDO1 which is more strictly regulated.^11^

IDO overexpression was described in several tumor localizations including the head and neck.^12^ Yet, very few studies have looked for IDO effects in NPC occurrence and disease progression. In NPC tissues, we previously observed the overexpression of IDO in 74% together with low T‐cell infiltration.^13^ In the present study, we aimed to investigate (i) the capacity of peripheral blood mononuclear cells (PBMC) to express IDO1 and IDO2, (ii) the plasma levels of Trp, Kyn, and the Kyn to Trp ratio (Kyn/Trp ratio), (iii) the correlation of plasma Kyn/Trp ratio with IDO gene expression in PBMC, (iv) the correlation of IDO gene expression and plasma Kyn/Trp ratio with clinicopathological parameters and patient survival, and (v) the correlation of interferon gamma (IFN‐γ), tumor necrosis factor‐α (TNF‐α), interleukin (IL)‐6, IL‐10 and IL‐17 cytokine levels with IDO in NPC.

## 2 MATERIALS AND METHODS

### 2.1 Subjects and sample collection

This prospective study was conducted on a total of 126 unrelated subjects belonging to the same population living in the middle coast of Tunisia. The study was approved by the Tunisian National Ethical Committee and informed consent was obtained from all enrolled individuals before participation. A total of 75 patients with confirmed NPC were recruited between 2012 and 2020 when they first presented at the Department of Cancerology and Radiotherapy of Farhat Hached University Hospital of Sousse, Tunisia. Patients presenting with any inflammatory disease, or receiving anticancer therapy or immunosuppressive medications, were excluded from the study. The age range of patients at the time of diagnosis was from 16 to 80 years (median, interquartile ranges [IQRs]: 47, 34–58). The age distribution of NPC rates is bimodal, with a first peak between the ages of 11 and 20 years and a second peak between the ages of 41 and 50 years. Respecting this distribution, young patients were defined as those aged 40 years or under while adult patients were over 40. The sex ratio was 2 (50 men and 25 women). The clinical stages ranged from I to IV according to the 7th edition of the International Union against Cancer/American Joint Committee on Cancer (UICC/AJCC) staging system.^14^ Without any selection, all patients presented with histologically confirmed UCNT. Among these, 70.3% were diagnosed at advanced stages (Stage III or IV) and 81.4% had lymph node metastasis. The follow‐up period ranged from 2 to 99 months (median, 36 months). During this period, 14 patients relapsed (local or distant recurrence) and 27 patients died due to nasopharyngeal carcinoma. Patients whose cause of death remained unknown were excluded. In this study, patients' clinical stages were grouped based on disease severity (I–II–III and IV). Demographic and clinical features of this cohort are detailed in Table 1. For tumor size, lymph node metastasis, distant metastasis and clinical stage, the sum does not equal the total due to unavailable data for one patient. The 51 healthy blood donors without any personal or family history of cancer or any inflammatory disease were recruited as controls from the Department of Hematology of Fattouma Bourguiba University Hospital of Monastir, Tunisia. Venous blood samples were collected from patients with NPC and healthy individuals into BD Vacutainer EDTA tubes. After centrifugation, the plasma was immediately separated and PBMC were isolated by Ficoll density gradient centrifugation. Each sample was stored at −80°C until use.

**Table 1:** Demographic and clinical characteristics of nasopharyngeal carcinoma patients

Characteristics	Patients (%)
Total	75 (100)
Age at diagnosis	
≤40	26 (34.7)
>40	49 (65.3)
Gender	
Men	50 (66.7)
Women	25 (33.3)
Tumor size^a^	
T1–T2	44 (58.7)
T3–T4	30 (40)
Lymph node metastasis^a^	
N0	13 (17.3)
N+	61 (81.4)
Metastasis^a^	
M0	70 (93.3)
M+	4 (5.4)
Clinical stage^a^	
I–II–III	45 (60)
IV	29 (38.7)
Recurrence	
No	61 (81.3)
Yes	14 (18.7)
Death	
No	48 (64)
Yes	27 (36)

^a^ The sum does not equal the total due to unavailable data.

### 2.2 Quantitative analysis of IDO1, IDO2, and IFN‐γ mRNA expression using real‐time PCR

Total RNA was extracted from isolated PBMC of 48 NPC patients and 43 healthy controls using the RNeasy Mini Kit with DNase digestion (Qiagen) and cDNA was synthesized using the iScript cDNA synthesis kit (Bio‐Rad) according to the manufacturer's instructions. The quantitative real‐time polymerase chain reaction (qPCR) assays were performed in duplicate on an iQ5 real‐time PCR detection system (Bio‐Rad) using SYBR Green Supermix (Bio‐Rad). IDO1, IDO2, and IFN‐γ mRNA expression were quantified and normalized to the expression of the reference gene, Glyceraldehyde‐3‐Phosphate Dehydrogenase (GAPDH), using specific primers.^15^ The thermal cycling of the real‐time PCR reaction was initiated by a denaturation step at 95°C for 10 min, followed by 40 cycles of denaturation at 95°C for 15 s and annealing primers at 58.2°C for 1 min. The standard curves were generated for each gene and the amplification efficiency was 90%–105%. The relative expression levels were calculated using the 2^‐∆Ct^ method and the relative changes in expression levels were determined using the 2^‐ΔΔCt^ method.

### 2.3 Measurement of plasma Kyn/Trp ratio using high‐performance liquid chromatography

Plasma concentrations of Trp and Kyn were measured using a validated high‐performance liquid chromatography (HPLC) in 70 NPC patients and 50 healthy controls. Briefly, 250 µl of plasma supplemented by 50 µl of 3‐nitro‐l‐tyrosine (50 µM), the internal standard, were deproteinized with 60 µl of trichloroacetic acid (30% w/v). After vortexing, precipitated protein was removed by centrifugation at 13,000 rpm for 15 min and 100 µl of the supernatant was injected into the HPLC system. The chromatographic separation was performed at 30°C using a C18 column (150 mm × 4.6 mm; 5 µm) and a mobile phase consisted of 15 mM phosphate buffer (pH 3.5) and 10.6% acetonitrile at a flow rate of 1.2 ml/min. Detection was carried out by a spectrophotometric detector at wavelengths of 360 nm from 0 to 3 min for Kyn and 280 nm from 3 to 8 min for Trp. Trp and Kyn concentrations were expressed as µM and the Kyn to Trp ratio was given as µM/mM.

### 2.4 Measurement of cytokines by enzyme‐linked immunosorbent assay

IFN‐γ, TNF‐α, IL‐6, IL‐10, and IL‐17 concentrations were measured in the plasma of 67 NPC patients and 33 healthy controls using ELISA kits (R&D Systems), according to the manufacturer's instructions. The minimum detectable levels were 8 pg/ml (IFN‐γ), 1.6 pg/ml (TNF‐α), 4.7 pg/ml (IL‐6), 3.9 pg/ml (IL‐10), and 15 pg/ml (IL‐17A). When the values were below the detection threshold, the minimum detectable level was assigned.

### 2.5 Statistical analysis

All data were analyzed using SPSS 22.0 statistical software (SPSS Inc.) and *p* value < .05 was considered significant. As not normally distributed, the data were presented as median (IQR) and nonparametric tests were employed. For continuous variables, Mann–Whitney *U* test was used to compare data between two independent groups and Spearman's rank correlation test was used to assess the direction and strength of the relationship.

Receiver operating characteristic (ROC) analysis was performed to set optimal cutoff values and divide patients into two subgroups “low versus high.” In the case of mRNA expression (IDO and IFN‐γ), no discrimination cutoff value was found and thus the median values were used as cutoff. A subgroup is considered as “low” when continuous variables are below or equal to the cutoff value and as “high” when continuous variables are above the cutoff value. The univariate and multivariate logistic regression analyses were used to determine the association between plasma Kyn/Trp ratio, IDO1, IDO2, and IFN‐γ mRNA expression, and clinicopathological features. Odds ratio (OR) and 95% confidence intervals (95% CI) were calculated.

Disease‐specific survival (DSS), disease‐free survival (DFS), and metastasis‐free survival (MFS) were analyzed using the Kaplan–Meier method for 5 years and compared with the log‐rank test. DSS was defined as the date of diagnosis until death due to nasopharyngeal carcinoma or last date of follow‐up. DFS was defined as the date of diagnosis until first recurrence, metastasis or the last date of follow‐up. MFS was defined as the date of diagnosis until first metastasis or last date of follow‐up. The univariate and multivariate Cox regression analyses were used to assess the impact of clinicopathological features and plasma Kyn/Trp ratio on survivals. Hazard ratio (HR) and 95% CI were calculated.

## 3 RESULTS

### 3.1 IDO1, IDO2, and IFN‐γ mRNA expression in peripheral blood mononuclear cells

**Figure 1:**
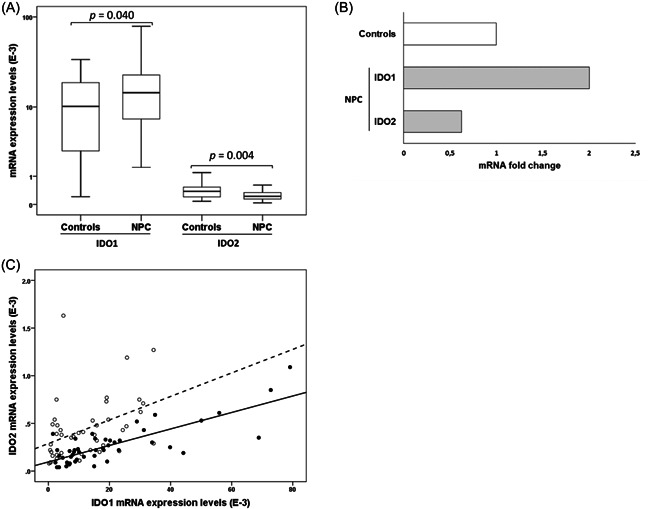
IDO1 and IDO2 mRNA expression in NPC patients. (A) IDO1 and IDO2 mRNA expression in nasopharyngeal carcinoma of healthy controls (*n* = 43) and NPC patients (*n* = 48), (B) Fold change (2^−ΔΔCt^) of IDO1 and IDO2 mRNA expression in NPC patients' relative to normalized Ct values of controls and (C) Correlation between IDO1 and IDO2 mRNA expression levels in controls (○, ‐‐) (*r* = .506, *p* < .001) and NPC patients (●, ─) (*r* = .691, *p* < .0001). E^−3^: ×10^−3^. mRNA, messenger RNA; NPC, nasopharyngeal carcinoma

IDO1 and IDO2 mRNA expression levels were quantified in PBMC of patients and controls using qPCR. The median ∆Ct values of IDO1 and IDO2 gene expression in controls were 6.62 (IQR: 5.71–8.53) and 11.35 (IQR: 10.06–12.28), respectively. The corresponding values in NPC patients were 6.09 (IQR: 5.42–7.14) and 12.17 (IQR: 11.52–12.80), respectively. IDO1 mRNA expression was significantly higher, whereas IDO2 mRNA level was significantly lower in NPC patients than in controls (*p* = .040 and *p* = .004, respectively) (Figure 1A). Fold change analysis showed that, compared to controls, IDO1 mRNA level increased by 2‐fold while IDO2 mRNA level decreased by 0.37‐fold in NPC patients (Figure 1B). When compared, IDO1 mRNA expression was greater than that of IDO2 about 27‐fold in controls and 67‐fold in NPC patients. The Spearman's rank correlation test showed a significant correlation between IDO1 and IDO2 mRNA expression in both controls and NPC patients (*r* = .506; *p* < .001 and *r* = .691; *p* < .0001, respectively) (Figure 1C). The IFN‐γ mRNA expression level was also quantified in PBMC of patients and controls. The quantification of IFN‐γ mRNA expression did not show a significant difference between NPC patients and controls.

### 3.2 Plasma Kyn/Trp ratio

**Figure 2:**
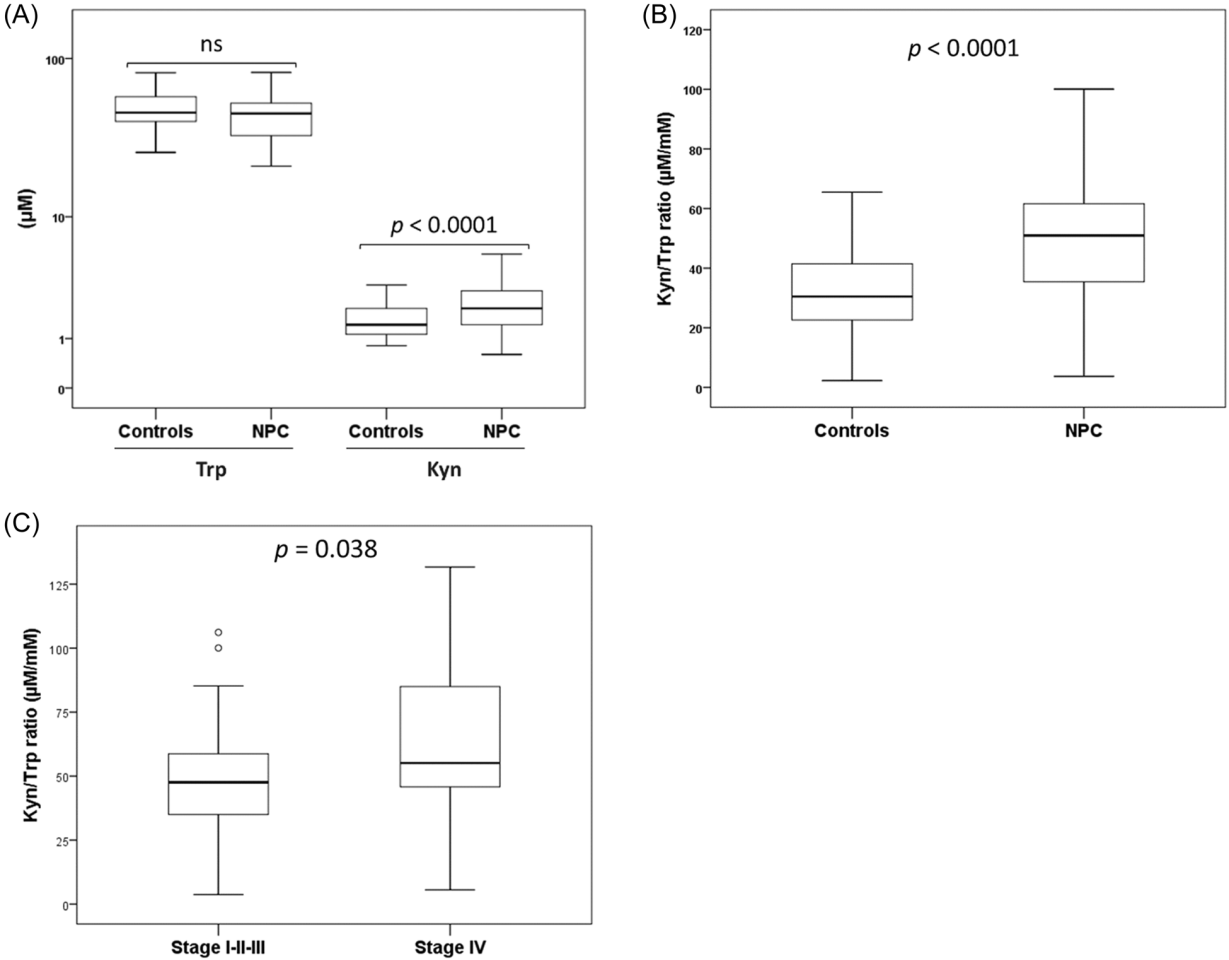
Plasma Kyn/Trp ratio in NPC patients. (A) Plasma Trp and Kyn levels, (B) Kyn/Trp ratio in healthy controls (*n* = 50) and NPC patients (*n* = 70) and (C) Kyn/Trp ratio according to clinical stage. Kyn, kynurenine; NPC, nasopharyngeal carcinoma; ns, not significant; Trp, tryptophan.

Plasma Trp and Kyn levels were determined by HPLC and the Kyn/Trp ratio was calculated. The median value of plasma Trp and Kyn levels and Kyn/Trp ratio in controls were 46.30 µM (IQR: 40.62–58.57), 1.43 µM (IQR: 1.11–2.05), and 30.47 µM/mM (IQR: 22.60–41.57), respectively. The corresponding values in NPC patients were 45.73 µM (IQR: 33.31–53.22), 2.05 µM (IQR: 1.43–2.92), and 50.96 µM/mM (IQR: 35.34–61.85), respectively. Plasma Kyn level and Kyn/Trp ratio were significantly higher in NPC patients than in controls (*p* < .0001 for both) (Figure 2A,B). As shown in Table 2, and according to the clinicopathological parameters of NPC at diagnosis, plasma Kyn/Trp ratio was significantly associated with younger age (≤ 40), larger tumor size (T3–T4), distant metastasis (M+), and advanced clinical stage IV (Figure 2C).

**Table 2:** Univariate logistic regression analysis of plasma Kyn/Trp ratio in NPC patients

Characteristics	Patients (%)	Kyn/Trp ratio^a^		OR (CI 95%)	*p* value
		Low	High		
Total	70 (100)	53	17		
Age at diagnosis					
>40	45 (64.3)	38	7	1	
≤40	25 (35.7)	15	10	3.62 (1.16–11.27)	.026*
Gender					
Women	23 (32.9)	19	4	1	
Men	47 (67.1)	34	13	1.81 (0.52–6.36)	.351
Tumor size					
T1–T2	41 (59.4)	36	5	1	
T3–T4	28 (40.6)	17	11	4.66 (1.40–15.53)	.012*
Lymph node status					
N0	12 (17.4)	9	3	1	
N+	57 (82.6)	44	13	0.89 (0.21–3.76)	.670
Metastasis					
M0	65 (94.2)	52	13	1	
M+	4 (5.8)	1	3	12 (1.15–124.99)	.038*
Clinical stage					
I–II–III	42 (60.9)	37	5	1	
IV	27 (39.1)	16	11	5.09 (1.52–17.04)	.008**
IDO1 mRNA^a^					
Low	24 (50)	15	9	1	
High	24 (50)	22	2	0.15 (0.03–0.80)	.026*
IDO2 mRNA^a^					
Low	26 (54.2)	18	8	1	
High	22 (47.8)	19	3	0.36 (0.08–1.55)	.169
IFN‐γ mRNA^a^					
Low	24 (50)	17	7		
High	24 (50)	20	4	0.49 (0.12–1.95)	.308

Abbreviations: CI, confidence interval; IFN‐γ, interferon gamma; Kyn, kynurenine; mRNA, messenger RNA; NPC, nasopharyngeal carcinoma; OR, odds ratio; Trp, tryptophan.

* <0.05;

** <0.01.

^a^ Low and high mRNA levels are classified according to cutoff values.

### 3.3 IFN‐γ, TNF‐α, IL‐6, IL‐10, and IL‐17 cytokine levels

**Figure 3:**
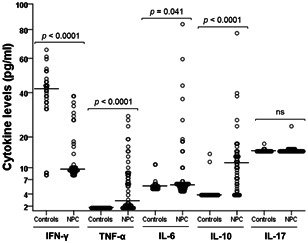
IFN‐γ, TNF‐α, IL‐6, IL‐10, and IL‐17 cytokine levels in healthy controls and NPC patients. IFN‐γ, interferon gamma; IL, interleukin; NPC, nasopharyngeal carcinoma; ns, not significant; TNF‐α, tumor necrosis factor‐α

To investigate the cytokine profile and its relationship with IDO expression and Kyn/Trp ratio in NPC, we examined the plasma levels of five different cytokines using ELISA. The IFN‐γ, TNF‐α, IL‐6, IL‐10, and IL‐17 median values in controls were 42.04 pg/ml (IQR: 35–46.24), 1.6 pg/ml (IQR: 1.6–1.6), 5.4 pg/ml (IQR: 5.07–5.72), 3.9 pg/ml (IQR: 3.9–3.91), and 15 pg/ml (IQR: 15–15.13), respectively. The corresponding values in NPC patients were 9.55 pg/ml (IQR: 8.95–12.58), 2.27 pg/ml (IQR: 1.87–8), 5.8 pg/ml (IQR: 5.10–6.10), 10.86 pg/ml (IQR: 5.42–13.97), and 15 pg/ml (IQR: 15–15.42), respectively. As shown in Figure 3, the plasma levels of IFN‐γ were significantly lower (*p* < .0001), whereas the plasma levels of TNF‐α, IL‐6 and IL‐10 were significantly higher (*p* < .0001, *p* = .041, and *p* < .0001, respectively) in NPC patients than in controls. Plasma IL‐17 levels did not show any significant difference between NPC patients and controls. Moreover, of all clinicopathological parameters, plasma levels of IFN‐γ, IL‐6, and IL‐10 were significantly higher in patients with distant metastasis compared to patients without distant metastasis (*p* = .039, *p* = .036, and *p* = .014, respectively). IFN‐γ plasma levels were also associated with advanced clinical stage (IV) (*p* = .007) (Data not shown).

### 3.4 The relationship between IDO1, IDO2, IFN‐γ mRNA expression, plasma Kyn/Trp ratio, and cytokine levels

The univariate and multivariate analysis and Spearman's rank correlation test were carried out to investigate the relationship between IDO1, IDO2 mRNA expression in PBMC, plasma Kyn/Trp ratio, and plasma cytokine levels. First, the univariate analysis confirmed that IDO1 and IDO2 mRNA expression were significantly associated in NPC patients (*p* = .001; OR [95% CI] = 11.54 [2.87–46.40]) and this association was independent as shown by the multivariate analysis (*p* = .002; OR [95% CI] = 12.30 [2.57–58.94]). Regarding these observations and the results of the gene expression, IDO1 was obviously the most expressed and therefore the most active isoform. Here, we only considered IDO1 mRNA expression in relationship with plasma Kyn/Trp ratio and cytokine levels.

The plasma Kyn/Trp ratio in IDO1‐high mRNA expression patients (median = 49.77; IQR: 34.74–58.72) was significantly lower than in IDO1‐low mRNA expression patients (median = 54.92; IQR: 35.36–89.71) (Table 2). Based on the patient's clinical stage groups, the Spearman's rank correlation test showed a positive correlation between IDO1 mRNA expression and plasma Kyn/Trp ratio (*r* = .373; *p* = .030) among patients with an earlier clinical stage (defined as clinical Stage I–II–III) (Table 3). However, a negative correlation between IDO1 mRNA expression and plasma Kyn/Trp ratio (r = −0.296; *p* = .096) was shown among patients with a later clinical stage (defined as clinical Stage IV) (Table 3). According to cytokines, a positive significant correlation between TNF‐α and Kyn (r = .364; *p* = .040), and a positive but not significant correlation between IFN‐γ and Kyn (*r* = .067; *p* = .393) were observed in patients with later clinical stage. In this patient group, a significant positive correlation was also observed between mRNA expression of IFN‐γ and mRNA expression of IDO1 in PBMC (*r* = .478; *p* = .016). Besides, IL‐6 and IL‐10 positively correlated with Kyn/Trp ratio in patients with later stage (*r* = .188; *p* = .184 and *r* = .266; *p* = .129, respectively). Conversely, negative correlations between TNF‐α or IFN‐γ and Kyn (*r* = −0.194; *p* = .125 and *r* = −0.049; *p* = .399, respectively) and a negative significant correlation between TNF‐α and mRNA expression of IDO1 in PBMC (*r* = −0.364; *p* = .040) were observed in patient with earlier stages (Table 3). Moreover, TNF‐α and IFN‐γ were negatively correlated whatever the disease stage (*r* = −0.316; *p* = .045 (Stage I–II–III); *r* = −0.457; *p* = .024 [Stage IV]).

**Table 3:** Correlation coefficients between plasma Kyn/Trp ratio, IDO1 mRNA expression, and cytokine levels in NPC patients

	Earlier stage (I–II–III)			Later stage (IV)		
	Kyn	Kyn/Trp ratio	IDO1 mRNA	Kyn	Kyn/Trp ratio	IDO1 mRNA
Kyn/Trp ratio	0.756**	‐	0.373*	0.744**	‐	−0.296
IFN‐γ mRNA	0.070	0.041	0.201	0.239	−0.081	0.478*
IFN‐γ (pg/ml)	−0.049	−0.085	0.268	0.067	0.219	−0.136
TNF‐α (pg/ml)	−0.194	−0.032	−0.364*	0.364*	0.214	−0.275
IL‐6 (pg/ml)	0.012	0.045	0.240	0.051	0.188	−0.466*
IL‐10 (pg/ml)	−0.192	−0.049	0.174	0.359	0.266	−0.502*
IL‐17 (pg/ml)	−0.172	−0.137	−0.300	−0.290	−0.369	0.095

Abbreviations: IFN‐γ, interferon gamma; IL, interleukin; Kyn, kynurenine; NPC, nasopharyngeal carcinoma; ns, not significant; mRNA, messenger RNA; TNF‐α, tumor necrosis factor‐α; Trp, tryptophan.

* *p* < .05,

** *p* < .0001 (unilateral Spearman's correlation).

According to a multivariate model including as covariates IDO1 mRNA expression and variables with a significant *p* value in the univariate analysis, plasma Kyn/Trp ratio was independently associated with the patient's age at diagnosis and clinical stage (Table 4).

**Table 4:** Multivariate logistic regression analysis of plasma Kyn/Trp ratio in NPC patients

	OR (CI 95%)	*p* value
Age at diagnosis _(≤40 vs. >40)_	6.23 (1.60–24.30)	.008
Clinical stage _(IV vs. I–II–III)_	7.28 (1.84–28.82)	.005

Abbreviations: CI, confidence interval; Kyn, kynurenine; NPC, nasopharyngeal carcinoma; OR, odds ratio; Trp, tryptophan.

### 3.5 Survival analysis and prognostic significance of plasma Kyn/Trp ratio

**Figure 4:**
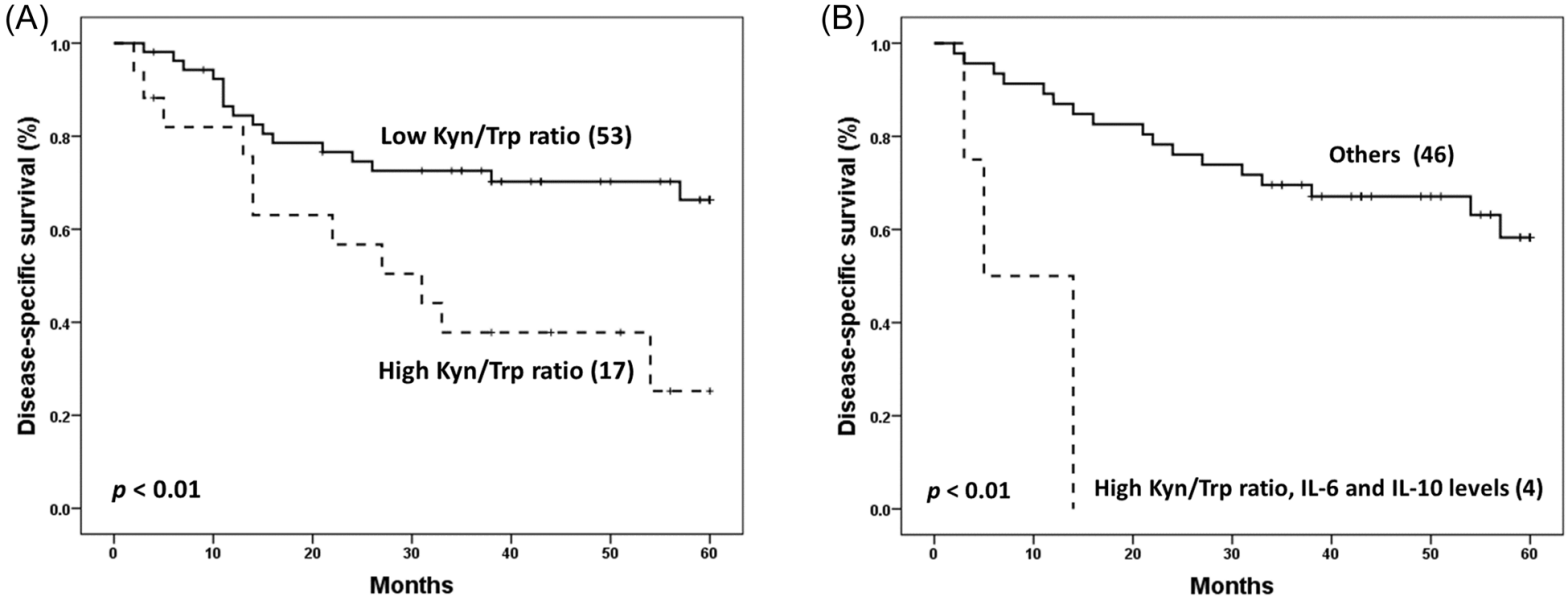
Disease‐specific survival analysis curves of NPC patients according to (A) plasma Kyn/Trp ratio (*n* = 70) and (B) combined plasma Kyn/Trp ratio with plasma levels of IL‐6 and IL‐10 (*n* = 50); Low and high designations are according to cutoff values. IL, interleukin; Kyn, kynurenine; NPC, nasopharyngeal carcinoma; Trp, tryptophan

The prognostic significance of Kyn/Trp ratio in NPC was first assessed by the analysis of their relationship with survival (DSS, DFS, and MFS). Patients with a high plasma Kyn/Trp ratio (more than 62.11 µM/mM) had a significantly lower 5‐year DSS (25.2%) than patients with low plasma Kyn/Trp ratio (66.3%) (*p* < .01) (Figure 4A). According to cytokines, all patients simultaneously carrying a high plasma Kyn/Trp ratio and high IL‐6 and IL‐10 plasma levels at diagnosis died 14 months later (*p* < .01) (Figure 4B).

Subsequently, univariate and multivariate Cox regression proportional hazard analyses were used to assess the impact of clinicopathological parameters and Kyn/Trp ratio on survival. The univariate analysis showed that gender (*p* = .009; HR = 4.94), tumor size (*p* = .031; HR = 2.31), distant metastasis (*p* < .001; HR = 13.44), clinical stage (*p* = .016; HR = 2.57), and plasma Kyn/Trp ratio (*p* = .008; HR = 2.80) were significant prognostic factors for a worse DSS (Table 5). Plasma Kyn/Trp ratio together with clinicopathological parameters with a significant *p* value in the univariate analysis were included in the multivariate analysis. Therefore, we showed that gender (*p* = .012; HR = 4.70) and plasma Kyn/Trp ratio (*p* = .015; HR = 2.62) were independent prognostic factors for a worse DSS (Table 5). This significant gender disparity is not a specific feature of the present study, but exists in NPC across several populations.^16^, ^17^

**Table 5:** Univariate and multivariate analysis with Cox proportional hazards model for disease‐specific survival (DSS)

	Univariate analysis			Multivariate analysis		
	HR	95% CI	*p* value	HR	95% CI	*p* value
Age at diagnosis _(≤40 vs. >40)_	1.10	0.49–2.45	.813	ni		
Gender _(Men vs. women)_	4.94	1.49–16.43	.009	4.70	1.41–15.66	.012
Tumor size _(T3–T4 vs. T1–T2)_	2.31	1.08–4.96	.031	ns		
Lymph node _(N+ vs. N0)_	1.28	0.44–3.71	.646	ni		
Metastasis _(M+ vs. M0)_	13.44	4.14–43.63	<.001	ns		
Clinical stage _(IV vs. I–II–III)_	2.57	1.19–5.54	.016	ns		
Kyn/Trp ratio _(High vs. low)_	2.80	1.29–6.06	.008	2.62	1.20–5.71	.015

*Note*: a: Low and high Kyn/Trp ratio are classified according to cutoff value.

Abbreviations: CI, confidence interval; HR, hazard ratio; Kyn, kynurenine; ni, not included in multivariate analysis; ns, nonsignificant; Trp, tryptophan.

## 4 DISCUSSION

NPC is a head and neck cancer with distinct biological and clinical features. One of the most crucial NPC traits is its high metastatic ability preventing survival improvement. Immune escape constitutes now an important hallmark of cancer and Trp oxidative breakdown, through the Kyn pathway, plays an important role in immune regulation. IDO which is one of the key enzymes in this pathway exerts immunosuppressive functions through Trp depletion and Kyn production.^18^ Since enzymatic activity of IDO is involved in the first and rate‐limiting step of the catabolism of Trp to Kyn and their metabolites, Kyn/Trp ratio evaluation was considered in several studies as an alternate for IDO activity estimation. Increased Kyn/Trp ratio, was found in several cancers such as melanoma, leukemia, lung, and cervical cancers.^19^, ^20^, ^21^, ^22^, ^23^ In NPC patients, the plasma Kyn/Trp ratio measured at time of diagnosis was associated with advanced cancer status. Similarly, in untreated patients with lung and cervical cancers, an increased Kyn/Trp ratio was associated with more advanced stage.^21^, ^22^, ^23^ We further demonstrated that plasma Kyn/Trp ratio assessed at time of diagnosis was an independent prognostic factor for a worse DSS of NPC patients despite treatment. Recently, in metastatic non‐small cell lung cancer, renal cell carcinoma, and head and neck squamous cell carcinoma treated with anti‐PD‐1, high serum Kyn/Trp ratio was associated with shorter overall survival.^24^ Altogether, these observations strongly support that, at time of diagnosis, plasma Kyn/Trp ratio measurement could be an indicator for NPC progression, and could be used to predict survival outcome among treated patients. Moreover, IDO seems to belong to systemic immunosuppressive actors. In NPC context, IL‐6 and IL‐10 levels, which are cytokines known to exert directly or indirectly immunosuppressive functions, act together with IDO to aggravate and shorten the NPC patient's survival to approximately 1 year. The interplay between immunosuppressive actors has also been demonstrated in the peripheral blood of melanoma patients, where IDO was linked with increased PD‐L1 + cytotoxic T‐cells which are in turn associated with increased CTLA‐4 expression by Tregs and monocytic myeloid‐derived suppressor cell (mMDSC) levels.^25^ Thus, plasma Kyn/Trp ratio evaluation could be more relevant when associated with other markers of immunosuppression.

In NPC, IDO expression has only been characterized in NPC tissue, which was detected in tumor cells, tumor‐infiltrating immune cells, and stromal macrophages.^13^, ^26^, ^27^, ^28^ However, very few studies investigated IDO expression by circulating immune cells from cancer patients. Approximately 3% of PBMC, predominantly plasmacytoid dendritic cells (pDCs) and monocytes constitutively express IDO.^29^ IDO expression by circulating pDCs and mMDSC has been demonstrated in melanoma to correlate with higher serum Kyn/Trp ratio.^25^ Herein, we investigated mRNA expression of the two *IDO* gene isoforms (IDO1 and IDO2) in PBMC and their relationship with plasma Kyn/Trp ratio in NPC patients. IDO1 mRNA expression was two‐fold upregulated and IDO2 mRNA expression was slightly downregulated in PBMC of NPC patients compared to controls. While, the mRNA expression level of IDO1 was greater than that of IDO2, IDO1 and IDO2 mRNA expression were significantly correlated, in both NPC patients and controls. This suggests that the functional IDO activity could result more from IDO1 activity than from IDO2. Although IDO2 catalyzes the same reaction as IDO1 and are genetically linked, the two enzymes have different expression and regulation patterns and distinct biological functions.^11^, ^30^ IDO2, which normally has a very restricted level of expression, has been found to be downregulated in cervical cancer compared to normal tissue.^31^ In the same study, Kyn/Trp ratio in cervical cancer tissue was correlated with IDO1 mRNA expression and not with IDO2 indicating that the increased IDO activity is due to IDO1 activity rather than IDO2. Overall, IDO1 seems to be the most biologically relevant Trp‐degrading enzyme in NPC compared to IDO2. In NPC patients presenting earlier clinical stage, a positive correlation between plasma Kyn/Trp ratio and IDO1 mRNA expression was found. Inversely, in patients with a later clinical stage, a negative correlation between plasma IDO1 mRNA expression and Kyn/Trp ratio was observed. One explanation of these results is that PBMC could express IDO at the promotion of the disease and contribute to the plasma IDO activity, later when the disease progresses, the tumor tissue could be the main source of IDO. Thus, the metabolic activity of IDO1‐expressing PBMC could be involved in NPC promotion rather than NPC progression. In the NPC tumor microenvironment, IDO expression has been observed in tumor cells as well as in the tumor‐surrounding stroma, which could enhance Kyn concentrations in the peripheral blood.^13^

Since IDO is under immunological control via the activation and the inhibition effects of cytokines, it is reasonable to assume that any cytokine production change would affect IDO expression. IDO expression is induced by various immunogenic molecules notably pro‐inflammatory cytokines, such as IFN‐γ, IL‐6, IL‐1β, and TNF‐α. Moreover, once induced, IDO expression can be maintained long‐term by tolerogenic signals, such as the Kyn‐AhR pathway.^32^ In our study, we looked for changes in plasma levels of pro‐inflammatory cytokines that could affect IDO gene expression and Kyn levels. Among patients with stage IV, we observed a positive significant correlation between TNF‐α and Kyn, and a positive but not significant correlation between IFN‐γ and Kyn. In this patient group, significant positive correlation was also noticed between mRNA expression of IFN‐γ and mRNA expression of IDO1 in PBMC. Conversely, in patient with lower stages, we observed negative correlations between TNF‐α or IFN‐γ and Kyn and a negative significant correlation between TNF‐α and mRNA expression of IDO1 in PBMC. Moreover, TNF‐α but not IFN‐γ was increased in NPC patients and these cytokine levels were negatively correlated whatever the disease stage. Altogether, these results supported the idea that pro‐inflammatory cytokines including INF‐γ and TNF‐α, have IDO regulatory effects that impact disease progression. Herein and in previous studies plasma IL‐6 and IL‐10 were found to be elevated and correlated with poor NPC prognosis. Unlike patients with early stages, in patients with stage IV, both IL‐6 and IL‐10 positively correlate with Kyn/Trp ratio. In NPC, IL‐6 activates signal transducer and activator of transcription 3 (STAT3) and promotes the proliferation, migration, and invasion of cancer cells.^33^, ^34^ Litzenburger et al., stipulated that IL‐6 is involved in a transcriptional positive feedback loop that maintain IDO (IDO‐Ahr‐IL‐6‐STAT3) and also IL‐6 expression in human cancer.^35^ Moreover, IL‐10 and TGF‐β are found to be expressed in NPC cells where they play a significant role in the recruitment of immune cells from peripheral blood to ensure an immunosuppressive environment.^36^ IL‐10 in assistance with IDO has been associated with the development of tolerogenic DCs. Indeed, IL‐10 prevented IFN‐γ‐induced IDO downregulation in DCs during their maturation, resulting in sustained expression of functional IDO in mature DCs.^37^ The worst impact of the cooperation between these immunosuppressive molecules is supported by the short survival period for NPC patients with simultaneous high Kyn/Trp value and high levels of IL‐6 and IL‐10. Little is known about the exact role of IL‐17 cytokine in carcinogenesis and progression of NPC. Consistent with previous observations, no significant clinical disparities were observed between groups, thus IL‐17 plasma levels value in NPC remains unclear.^38^

Further studies are required to better understand the regulation of IDO expression and activity in NPC to prevent IDO‐mediated immune escape. In the context of immunotherapy, pharmacological inhibition of IDO was the focus of numerous studies. In preclinical models, it has been reported that IDO blockade potentiates the antitumor efficacy, when combined with immune checkpoint therapy and, DNA‐damaging chemotherapy and radiotherapy.^39^, ^40^ Recently, several Phase I/II clinical trials have assessed IDO inhibitors and showed promising results in multiple advanced tumors.^41^, ^42^, ^43^ Currently, at least eight small molecule IDO1 inhibitors are being assessed in clinical trials indicated by the clinical trial registry website ClinicalTrials.gov (https://clinicaltrials.gov).^44^

The limitation of this study was the relatively limited enrolled research samples as this was a prospective study. In addition, our data were only descriptive and IDO plays an immunosuppressive role in tumor through multiple signaling pathways. Further research works with larger sample are needed to find out the exact mechanism of IDO leading to NPC progression.

In conclusion, PBMC could participate in the establishment of the cancer disease through an effective IDO expression, especially for early diagnosed NPC patients. Moreover, this study provides evidence for the benefit of measuring plasma Kyn/Trp ratio at diagnosis. Together with plasmatic immunosuppressive markers such as IL‐6 and IL‐10, Kyn/Trp ratio could provide a reliable prognosis for better management of NPC patients. A better understanding of the IDO pathway inside the tumor and also at the periphery may be useful to set up well‐personalized and more relevant therapeutic strategies.

## AUTHOR CONTRIBUTIONS

Elham Hassen and Sameh Souissi conceived the study. Sameh Souissi, Yosra Macherki, Ahlem Ben‐Haj‐Ayed, Yasmine Remadi, Mohsen Hassine, Noureddine Bouaouina, and Abdelfattah Zakhama helped in sample and data collection. Sameh Souissi, Randa Ghedira, and Sallouha Gabbouj carried out the molecular analysis. Sameh Souissi, Randa Ghedira, Zohra Chadli, and Karim Aouam carried out the HPLC analysis. Sameh Souissi and Imen Sfar carried out ELISA analysis. Elham Hassen, Sameh Souissi, Yosra Macherki, and Noureddine Bouaouina generated and analyzed the data. Elham Hassen and Sameh Souissi discussed the results and wrote the manuscript.

## CONFLICTS OF INTEREST

The authors declare no conflicts of interest.

## ETHICS STATEMENT

The study was approved by the Tunisian National Ethical Committee. All procedures performed in the study has been performed in accordance with the ethical standards of the institutional or national research committee and with the 1964 Helsinki declaration and its later amendments or comparable ethical standards.

## Data Availability

The data that support the findings of this study are available on request from the corresponding author.
